# IgG Serum Antibodies to *Shigella sonnei* Lipopolysaccharide Are Inversely Associated with the Incidence of Culture-Proven *S. sonnei* Shigellosis in Israeli Children and Adolescents

**DOI:** 10.3390/vaccines12030239

**Published:** 2024-02-25

**Authors:** Valeria Asato, Ravit Bassal, Shiri Meron-Sudai, Sophy Goren, Lital Keinan-Boker, Calman A. MacLennan, Dani Cohen

**Affiliations:** 1 Department of Epidemiology and Preventive Medicine, School of Public Health, Faculty of Medicine, Tel Aviv University, Tel Aviv 6997801, Israel; valeria.asato@gmail.com (V.A.); ravit.bassal@moh.health.gov.il (R.B.); shiri.meron.sudai@gmail.com (S.M.-S.); sophyg@tauex.tau.ac.il (S.G.); 2 Israel Center for Disease Control, Ministry of Health, Chaim Sheba Medical Center, Tel-Hashomer, Ramat-Gan 52621, Israel; lital.keinan2@moh.gov.il; 3 Bill & Melinda Gates Foundation, 62 Buckingham Gate, London SW1E 6QW, UK; calman.maclennan@gatesfoundation.org; 4 Jenner Institute, Nuffield Department of Medicine, University of Oxford, Oxford OX1 2JD, UK

**Keywords:** IgG, *Shigella*, shigellosis, incidence, children, Israel

## Abstract

Background: *Shigella* is a leading cause of moderate-to-severe diarrhea globally, with young children most affected. The burden of shigellosis drops increasingly with age, inferring the acquisition of natural immunity. We tested the hypothesis that IgG antibodies elicited against *Shigella* O-specific polysaccharide (O-SP) are correlates of age-acquired immunity. Objectives: We examined levels and determinants of serum IgG to *S. sonnei* LPS and the association with the incidence of *S. sonnei* shigellosis in Israeli children and adolescents. Methods: We analyzed 1096 serum samples from 0- to 19-year-olds collected in 2008–2015 for IgG anti-*S. sonnei* LPS levels by ELISA. Corresponding age-specific incidences of culture-proven *S. sonnei* shigellosis from 2008 to 2015 were obtained. We compared ecologically IgG levels, prevalence above a proposed protective threshold, and *S. sonnei* shigellosis incidence. Results: In a multivariable analysis model, children aged 1–4, 5–14, and 15–19 years were 6.71, 27.68, and 48.62 times more likely to have IgG anti-*S. sonnei* LPS above the threshold than those aged < 1 year, respectively (*p* < 0.001). Infants 0–3 months old had relatively high IgG anti-*S. sonnei* LPS levels of maternal origin that dropped thereafter. Children of low socioeconomic status had a 2.73 times higher likelihood of having IgG anti-*S. sonnei* LPS above the threshold (*p* < 0.001). A significant inverse correlation between age-specific IgG anti-*S. sonnei* LPS levels and *S. sonnei* shigellosis incidence was observed (Spearman rho= −0.76, *p* = 0.028). Conclusions: The study results support anti-*S. sonnei* LPS antibodies as correlates of protection that can inform *Shigella* vaccine development.

## 1. Introduction

*Shigella* is a leading cause of moderate-to-severe diarrhea and diarrheal deaths worldwide. The greatest burden of shigellosis is in low- and middle-income countries (LMICs) with poor sanitation, resulting in approximately 250 million cases of disease and more than 200,000 deaths annually, mostly in children under 5 years of age [1]. In LMICs, children with shigellosis have an increased risk of persistent diarrhea, malnutrition, and linear growth faltering [2,3,4]. Around 1.5–2 million cases of shigellosis also occur annually in high-income countries (HICs), mostly among toddlers in crowded communities, among travelers visiting less developed regions, soldiers who serve under field conditions in endemic regions, and among men who have sex with men [1,5,6,7,8,9,10]. Considering the global burden of shigellosis and the emerging antimicrobial resistance of *Shigella* [11,12,13,14], a *Shigella* vaccine is of high priority [15,16,17,18,19]. 

The genus *Shigella* includes four serogroups or species: *S. dysenteriae* (divided into 15 serotypes), *S. flexneri* (composed of 15 serotypes), *S. boydii* (19 serotypes), and *S. sonnei* (1 single serotype), defined by conformational epitopes of the lipopolysaccharide (LPS) O-antigens [12]. *S. flexneri* and *S. sonnei* are responsible for around 90% of shigellosis cases [20,21]. *S. flexneri* is predominant in LMICs (in particular serotypes 2a and 6) while *S. sonnei* is the leading *Shigella* species in HICs [6,20]. An emerging trend has been documented in which *S. sonnei* is gradually replacing *S. flexneri* as the leading cause of shigellosis in countries undergoing economic development and improvements in sanitation and water quality [22,23,24].

Shigellosis is highly endemic in Israel though Israel is a HIC. *S. sonnei* is responsible for more than 85% of the cases of shigellosis in the general population (more than 95% among Jews), while *S. flexneri* and *S. sonnei* are similarly associated with shigellosis in the Israeli Arab subpopulation of Israel [6].

It is accepted that *Shigella* infection provides serotype-specific immunity for a relatively short duration of approximately 2 years [6,25], consistent with the O-specific polysaccharide (O-SP) antigen of *Shigella* species being the protective antigen. Serum IgG antibodies against *Shigella* O-SP have been proposed as a correlate of protection [26] and threshold levels of IgG anti-*S. sonnei* LPS predicting defined degrees of protection have been identified [16]. 

The incidence of shigellosis is highest in children under 5 years of age and drops thereafter, inferring an age-dependent acquisition of natural immunity. The goal of the present study was to test the hypothesis that IgG antibodies elicited against *Shigella* O-SP mediate the age-acquired immunity against homologous disease. To this end, we measured the level and the determinants of serum IgG antibodies to *S. sonnei* LPS and examined the association between the antibody levels and the incidence of culture-proven *S. sonnei* shigellosis (confirmed by a positive stool culture for *S. sonnei*) in Israeli children and adolescents. In the absence of information on both antibody levels and *S. sonnei* shigellosis status (positive or negative) at the individual level, we could determine ecological associations, namely correlations, between average levels of serum IgG antibodies against *S. sonnei* LPS and average incidences of culture-proven *S. sonnei* shigellosis across the various age groups within the entire study population and within population groups. 

## 2. Methods

### 2.1. Study Design and Study Population

#### 2.1.1. Study Design 

We employed a cross-sectional study design to measure the level and identify the determinants of serum IgG antibodies to *S. sonnei* LPS and drew ecological comparisons to examine the association between the antibody levels and the incidence of culture-proven *S. sonnei* shigellosis.

#### 2.1.2. Study Population

A stratified random sample of 1096 anonymized archived sera obtained between the years 2008 and 2015 from 0- to 19-year-old Jewish and Arab children and adolescent residents of Israel was drawn from the Israel National Sera Bank (INSB). Similar representation of both genders and Jewish and Israeli Arab population groups was targeted in the various age strata. The INSB, established by the Israel Center for Disease Control (ICDC) in 1997, monitors the immunity of the Israeli population against vaccine- and non-vaccine-preventable diseases of public health importance [27]. The serum samples for the INSB are collected from six laboratories and are all residual sera from healthy blood donors or individuals who have undergone routine or diagnostic blood tests excluding those related to immunological conditions. All serum samples at the INSB are anonymized, while demographic data such as sex, age, religion, nationality, and place of residence (at the town level) are retained for each sample [27]. 

The ecological association between IgG anti-*S. sonnei* LPS antibody levels and the incidence of *S. sonnei* shigellosis in Israeli children and adolescents was examined using data on age, sex, and population group-specific incidence of culture-proven cases of *S. sonnei* shigellosis (*n* = 11,319) collected in the years 2008–2015 through the Sentinel Laboratory-Based Surveillance Network of Bacterial Enteric Diseases (SLBSN), established by the ICDC, Israel Ministry of Health, in 1998 [6]. Monthly reports from five sentinel community microbiological laboratories, located across Israel and serving 30.8% of the Israeli population, provided information on positive stool culture samples for *Shigella* of patients with diarrhea, along with the phenotypic characterization of the isolates at the serotype level and patient demographic data. Age, sex, and population group-specific incidence data were calculated. The culture-proven cases of *S. sonnei* shigellosis served as the numerator, and the age, sex, and population group distribution of the catchment population of the laboratories, according to the Central Bureau of Statistics and Health Maintenance Organizations (HMOs) data, served as the denominator. The sampling frame of residual serum samples of the INSB is based on medical centers with similar geographic distribution and demographic coverage as the SLBSN sentinel labs and in part sharing the same location. The sampling frames and definition of variables related to the two surveillance systems did not change over the years 2008–2015. 

Socioeconomic status (SES) was determined using the Israel Central Bureau of Statistics classification of the residential SES rank at the level of city/town/village. Ranks are on a scale from 1 to 10, with lower ranks representing a lower SES. This is an aggregate SES score calculated using multiple socio-demographic and economic factors, including financial resources of the residents, housing conditions, motorization level, education, and employment profile [28]. Two SES were defined: low SES, including clusters 1 and 2, and middle and high SES, including clusters 3–10, respectively.

### 2.2. Serum Samples Handling and Laboratory Methods

A total of 500 microliter aliquots of all 1096 serum samples stored at −80 °C at the INSB were transferred frozen to the Research Lab at the Tel Aviv University School of Public Health, where they were maintained at −80 °C until tested.

Measurement of IgG anti-*S. sonnei* LPS antibodies: levels of serum IgG antibodies to *S. sonnei* LPS were measured by ELISA as previously described [29]. The LPS coating antigen used was a gift from the GSK Vaccines Institute for Global Health, Siena, Italy. It was extracted from the outer membrane vesicles of *S. sonnei* 53G DtolR virG::nadAB strain [30], adapting the Westphal procedure [31]. Briefly, 96-well microtiter plates (Corning) were coated with the *S. sonnei* LPS in carbonate buffer (pH 9.6 ± 0.05) for 1 h at 37 °C. Unbound sites were blocked with blocking buffer containing 0.5% bovine serum albumin (BSA) (Merck Millipore, Kankakee, IL, USA) and 0.5% Casein (Sigma Aldrich, Saint Louis, MO, USA) for 1 h at 37 °C. The wells were washed and tested samples were serially 2-fold diluted in blocking buffer, added to the wells, and incubated overnight (ON) at room temperature (RT). Plates were washed and alkaline phosphatase (AP)-conjugated goat antibody anti-human IgG (γ) (KPL, Sera Care, MA, USA) was added and incubated ON at RT. Thereafter, the wells were washed and phosphate substrate, para-nitrophenylphosphate (pNPP) one component (SouthernBiotech, Birmingham, AL, USA), was added and the plates were incubated in the dark for 15 min at RT. The reaction was stopped by the addition of 3 M NaOH. Absorbance (A) was measured at 405 nm using an ELISA plate reader (Multiskan FC, Thermo Scientific, Waltham, MA, USA). The results were expressed in ELISA units (EUs) related to a standard that was assigned a value of 100 units. The threshold of 4.5 EUs IgG antibodies to *S. sonnei* LPS was found to be associated with 50% protection against *S. sonnei* shigellosis, as previously reported [16]. 

### 2.3. Statistical Methods 

Descriptive statistics for the demographic characteristics of the participants were performed. Geometric Mean (GM) levels and 95% Confidence Intervals (CIs) were calculated for antibody levels after log transformations. Student’s *t*-test and one-way ANOVA were used to examine differences in the GM between the populations. 

The threshold level of 4.5 EUs was used to determine the prevalence of serum IgG antibodies to *S. sonnei* LPS (i.e., to signify the ‘presence’ of serum IgG to *S. sonnei* LPS equal to or above 4.5 EUs). Univariate and multivariate logistic regression analysis was performed. Unadjusted and adjusted odds ratios (ORs) and 95% CIs were used to determine the strength of association between the independent and dependent variables. Correlations were assessed using Spearman’s rank correlation coefficient. All statistical tests were interpreted two-tailed using a significance level (α) = 0.05. Data were analyzed utilizing the SPSS version 29 (IBM Corp., Armonk, NY, USA). 

## 3. Results

### 3.1. IgG Anti-Shigella LPS Antibody Levels and Correlates 

The characteristics of the study population are summarized in Table 1. 

Table 2 depicts the association between sociodemographic factors and IgG anti-*S. sonnei* LPS levels. The level of IgG anti-*S. sonnei* LPS antibodies increased constantly from the first year of life to adolescence by means of both GM values and proportion of prevalence in relation to the cutoff of antibody level defined (*p* for linear trend in prevalence < 0.001) (Table 2).

In infancy, there was a clear decay of IgG anti-*S. sonnei* LPS antibody levels (GM and prevalence) after 3 months of age toward the end of the first year of life. 

In a univariable logistic regression analysis, the likelihood to have antibodies above 4.5 EUs was 7.64-, 29.75-, and 52.99-fold higher for children aged 1–4, 5–14, and 15–19 years as compared to infants (reference age group), respectively (Table 2). The likelihood of having IgG anti-*S. sonnei* LPS equal or above the threshold level was 1.50-fold higher in females than in males, 1.97-fold higher in Arabs than in Jews, and 3.08-fold higher in children of low SES (clusters 1–2) compared to those of middle and high SES (clusters 3–10) (Table 2). 

Table 3 displays the results of the multivariable analysis. Age and population group maintained an independent and significant association with the prevalence of IgG anti-*S. sonnei* LPS above 4.5 EUs when these variables and sex were included in model 1. In model 2, children of low SES (clusters 1–2) had a 2.79-fold (95% CI: 1.78–4.38 *p* < 0.001) higher likelihood of the presence of IgG anti-*S. sonnei* LPS compared with children of middle and high SES (clusters 3–10) and the chance of having an IgG anti-*S. sonnei* LPS level of 4.5 EUs or above was significantly higher in all age groups older than 1 compared to the reference group of infants. 

When SES and population group were both added to the model (model 3), SES annulled the association between the Arab population group and the presence of IgG anti-*Shigella* LPS, showing that children of low SES (clusters 1–2) of any population group (Jews or Arabs) had a 2.73-fold higher likelihood of the presence of IgG anti-*Shigella* LPS at a level of 4.5 EUs or above compared with children of middle and high SES (clusters 3–10) (95% CI: 1.37–5.46, *p* = 0.004). 

### 3.2. IgG Anti-S. sonnei LPS Levels and an Ecological Inverse Association with S. sonnei Shigellosis 

**Figure 1:**
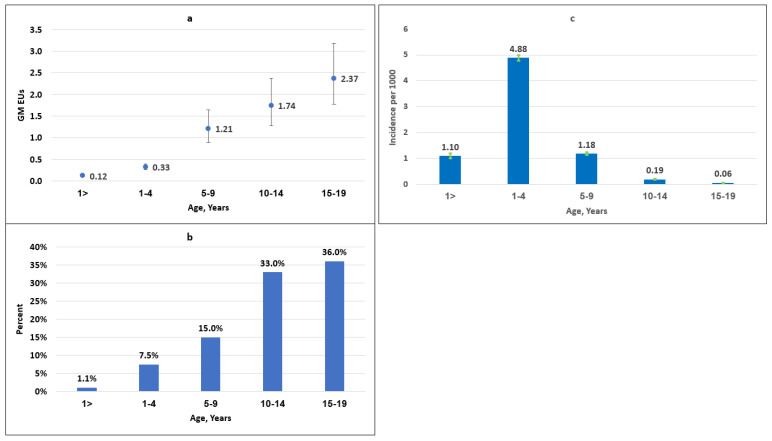
(**a**) Levels of serum IgG anti-*S. sonnei* LPS antibodies expressed as GM ELISA units of a standard (EUs) in ascending age groups of 1096 Israeli children and adolescents. Vertical lines indicate 95% CI. (**b**) Proportion of individuals with IgG anti-*S. sonnei* LPS levels ≥ 4.5 EUs in ascending age groups of 1096 Israeli children and adolescents. (**c**) Average annual incidence per 1000 of *S. sonnei* shigellosis in the general population served by sentinel laboratories in Israel stratified by the corresponding age groups, during 2008–2015 (based on 11,319 *S. sonnei* isolates). Vertical lines indicate 95% CI.

**Figure 2:**
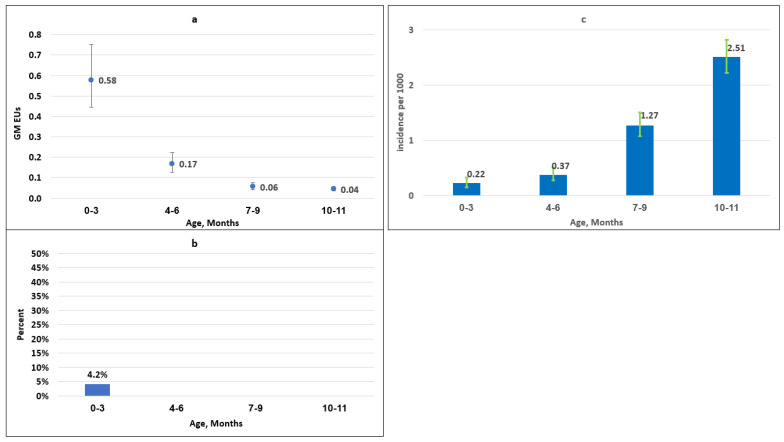
(**a**) Levels of serum IgG anti-*S. sonnei* LPS antibodies, expressed as EUs among 476 Israeli infants. Vertical lines indicate 95% CI. (**b**) Proportion of individuals with IgG anti-*S. sonnei* LPS levels ≥ 4.5 EUs among 476 Israeli infants. (**c**) Average annual incidence per 1000 of *S. sonnei* shigellosis in infants during 2008–2015 (based on 455 *S. sonnei* isolates). Vertical lines indicate 95% CI.

The average annual age-specific incidence rates of *S. sonnei* shigellosis followed a mirror-image curve for age groups 1 to 19 years to that displayed by antibody levels expressed in GMs or prevalence rates (Figure 1a–c and Supplementary Material, Table S1). The average age-specific annual incidences of culture-proven *S. sonnei* shigellosis in Israel, based on the SLBSN including 11,319 *S. sonnei* isolates between 2008 and 2015, were 4.88, 1.18, 0.19, and 0.06 per 1000 at ages 1–4, 5–9, 10–14, and 15–19, respectively. The corresponding IgG anti-*S. sonnei* LPS prevalence rates were 7.5, 15.0, 33.0, and 36.0, and the GM values were 0.33, 1.21, 1.74, and 2.38 (Figure 1a–c and Supplementary Material, Table S1). *S. sonnei* shigellosis incidence was also inversely associated with antibody levels in the first year of life. The higher levels of IgG antibodies to *S. sonnei* LPS found in the first trimester of life drastically decreased up to 12 months. The average annual *S. sonnei* shigellosis incidence rates per 1000 followed an opposite trend, increasing with the decay in the homologous antibody levels (0.22, 0.37, 1.27, and 2.51 cases per 1000 versus 4.2%, 0%, 0%, and 0% prevalence of IgG antibodies to *S. sonnei* LPS in age groups 0–3, 4–6, 7–9, and 10–11 months and GMs of 0.58, 0.17, 0.06, and 0.04) (Figure 2a–c and Supplementary Material, Table S2). There was a significant ecological correlation between the eight parallel GMs of IgG antibodies to *S. sonnei* LPS and *S. sonnei* incidence rates from birth to 19 years of age (Spearman rho = −0.76, *p* = 0.028) (Figure 1a–c and Figure 2a–c).

The proportion of individuals with IgG anti-*Shigella* LPS levels equal to or higher than 4.5 EUs and GM EU levels was overall lower in Jewish than in Arab children in contrast to the incidence rates, which were significantly higher among Jews than among Arabs. In both population groups, the increasing proportion of individuals with IgG anti-*Shigella* LPS levels above the cutoff (or GM EUs of specific IgG antibodies) in 1- to 19-year-old children correlated with decreasing incidence rates of *S. sonnei* shigellosis in these age ranges. A similar pattern was observed in infancy. There was an overall strong and significant inverse correlation between the 8 parallel GMs of IgG antibodies to *S. sonnei* LPS and *S. sonnei* incidence rates from birth to 19 years of age in Arabs (Spearman rho = −0.9, *p* < 0.001) and a similar trend in Jews, albeit not reaching statistical significance in Jewish children (Spearman rho = −0.571, *p* = 0.13) (Supplementary Material: Figures S1a–c and S2a,b). 

In a sub-analysis restricted to immunological and morbidity data of individuals living in the same region, we merged data on serum samples collected (*n* = 467) and cases of *S. sonnei* shigellosis identified (*n* = 2762) during 2008–2015 through the same medical center covering towns and settlements in the South of Israel (Supplementary Material, Figures S3a–c and S4a,b). The ecological inverse correlation between GM IgG anti-*S. sonnei* LPS and *S. sonnei* shigellosis incidences was consistent in the eight parallel age groups and statistically significant (Spearman’s rho = −0.714; *p* = 0.047).

The correlations found between GM or the prevalence of IgG anti-*S. sonnei* LPS levels according to the ≥4.5 EUs and alternative threshold EUs values and incidences of *S. sonnei* shigellosis are summarized in Table S7 in the Supplementary Material. GM as a continuous variable showed the highest and statistically significant inverse correlation (r = −0.762, *p* = 0.028) with the incidence rates determined in eight age groups. Prevalences of antibodies equal to or above the various thresholds all showed correlation trends ranging from r = −0.577 to r = −0.653 with the incidence rates, though not statistically significant.

## 4. Discussion 

In this study, we investigated levels and determinants of serum IgG antibodies to *S. sonnei* O-SP and the association of these antibodies with the incidence of *S. sonnei* shigellosis in Israeli children and adolescents. We found that age and SES were the main determinants of the prevalence of IgG antibodies to *S. sonnei* LPS. The clear pattern of age-dependent increase in IgG anti-*S. sonnei* LPS antibody level that was observed is most probably the result of repetitive natural symptomatic or asymptomatic exposures to *S. sonnei* LPS or to cross-reacting antigens of *Enterobacteriaceae.* Similar findings were previously reported concerning other *Shigella* serogroups in pediatric populations living in endemic countries. Antibody levels of *S. flexneri* 2a LPS increased with age in Chilean children, with the highest titers in the 15-year-old group [32]. In addition, significantly higher *S. flexneri* and *S. dysenteriae* type 1 LPS IgG serum levels were found in Bangladeshi children between 2.5 and 8 years compared with infants of 6–10 months [33].

The higher levels of IgG antibodies to *S. sonnei* LPS found in the first trimester of life and their further decay likely reflects the presence of antibodies of maternal origin after trans-placental transfer. A rapid decline in IgG antibody levels to *S. sonnei* after birth and undetectable maternal antibodies by 5 months of age was also noted in Vietnamese infants [34]. In Zambian infants, relatively high levels of IgG antibodies to *S. flexneri* 2a and *S. sonnei* LPS were measured at 6 and 14 weeks after birth. The GMTs declined at 12 months, although individual infants already developed a significant serologic response following exposure to *Shigella* antigens [35].

SES was the second strongest determinant of IgG anti-*Shigella* LPS prevalence, suggesting the high relative importance of living conditions, and particularly crowding, with respect to exposure to *S. sonnei*, which is responsible for more than 85% of cases of shigellosis in Israel [6]. Age, population group, and SES-associated levels of antibodies to *Shigella* antigens (LPS and invasive plasmid antigens) were previously reported in studies conducted in healthy Chilean and North American [32], and in Swedish, and Costa Rican [36] populations, and Israeli subpopulations [37].

The low infectious dose of *Shigella* spp. [38] allows the fecal–oral transmission of the microorganism through multiple routes, such as person-to-person, foodborne, waterborne, fly-borne, and fomite-borne, that are more common in communities of lower SES and with inadequate hygienic and sanitary facilities [37,39,40].

To assess whether IgG anti-*S. sonnei* LPS levels could serve as a correlate of protection, we analyzed data from two valuable Israeli databases: the INSB and the SLBSN. We established a significant ecological inverse correlation between IgG anti-*S. sonnei* LPS levels and the incidence of shigellosis caused by the homologous *Shigella* serotype in Israeli children and adolescents, supporting the definition of IgG anti-*S. sonnei* LPS as a correlate of protection. The negative correlations were consistent across different age groups and population groups (Israeli Arabs and Jews) and the IgG anti-LPS antibody levels were expressed as seroprevalence proportions with dichotomous (positive/negative) levels around a defined cutoff level. The cutoff level used (4.5 EUs) was previously validated as a threshold indicating 50% protection against *S. sonnei* shigellosis in children [16]. As previously proposed, a minimal protective level of serum IgG antibodies against *Shigella* O-SP, induced by infection or vaccination, can transudate into the intestinal lumen and lyse the invading *Shigella* organisms directly or through complement-dependent serum bactericidal activity conferring protection against homologous disease [41,42]. 

An overall lower incidence rate of shigellosis was observed in infants compared to older children, as also reported for other countries endemic for shigellosis, including LMICs [43]. This is probably related to a combined effect of a lower risk of exposure to *Shigella* in breastfed infants, together with the presence of placentally transferred maternal IgG antibodies to *Shigella* LPS [34,44], and possibly secretory IgA and IgG supplemented through breast-feeding. Though lower than later in childhood, the incidence of *S. sonnei* shigellosis in the first year of life increased gradually by month, being inversely associated with the decay in the levels of IgG anti-*S. sonnei* LPS antibodies. The short-term passive immune protection conferred by maternal antibodies was not replaced by a similarly effective immune response in infants. Due to the immaturity of the immune system, infants elicit a weak immune response after first antigen encounters without generating B cell memory [45,46] that subsequently develops after additional exposures. The highest incidence of *S. sonnei* shigellosis occurred in the 1–4 age group. Enhanced exposure due to poor hand hygiene, increased contact with feces in the toilet training phase, diaper practices of caregivers, and other specific risk factors in crowded daycares or households [6] coincides with the very low IgG anti-*S. sonnei* LPS levels that we detected in this age group. 

To the best of our knowledge, this is the first comprehensive *Shigella* analytical study to examine ecological associations between an immunological parameter, IgG anti-LPS antibodies in this case, and the incidence of shigellosis in the same general population of children and adolescents. A similar analytical approach was used in the seminal study of the 1960s showing the correlation between the age-related incidence of meningococcal disease in the United States and the prevalence of serum bactericidal activity against three pathogenic strains of *N. meningitidis* [47]. That was the breakthrough in understanding serum antibody protection and guiding the development of meningococcal vaccines [47].

Our study has several limitations. First, paired maternal and infant samples were not available to determine the magnitude of specific maternal antibodies transferred to the fetus and the waning of these antibodies in the infant’s serum. Second, considering the type of study that we conducted (ecological study), we could not evaluate the correlation between individual serum IgG antibodies anti-*S. sonnei* LPS observed with the protection against the homologous *Shigella* species. Third, the incidence of *S. sonnei* shigellosis could be underestimated, though without an expected differential impact on the findings of the study, considering that only cases of culture-proven *S. sonnei* shigellosis are counted by the SLSBN and other additional factors could reduce the yield of case detection. There might also be a differential utilization of medical services and health-seeking behavior concerning diarrheal diseases in the Jewish and Arab population groups, possibly leading to a greater underestimation of the burden of shigellosis in Israeli Arabs.

This study has several strengths. First, it includes a large number of sera of children of different ages, population groups, and socioeconomic and cultural backgrounds drawn from the INSB and evaluated in this study. Second, it utilizes multi-year incidence data made available for children from the same age range and population group by the national SLBSN. These two surveillance systems enabled the ecological analyses to be conducted.

## 5. Conclusions

Our study demonstrates that age and SES are significant determinants of IgG antibody levels in *S. sonnei* LPS. The ecological analysis indicates an inverse association between IgG anti-*S. sonnei* LPS levels and the incidence of homologous shigellosis. These findings are critical for refining host and *Shigella* species-related correlates of protection, which are important steps in the development and evaluation of *Shigella* vaccine candidates. Contemplating the availability of an efficacious *S. sonnei* vaccine in the next years, if immunization would be considered, the suitable timing for a first dose of vaccine would be at six to nine months of age.

## Figures and Tables

**Table 1:** Characteristics of the study population (n = 1096).

Characteristic	N (%)		
Age			
0–3 months	118 (10.8)		
4–11 months	358 (32.7)		
1–4 years	320 (29.2)		
5–14 years	200 (18.2)		
15–19 years	100 (9.1)		
Years, Mean (SD)	4.5 (5.4)		
Sex			
Males	575 (52.5)		
Females	521 (47.5)		
Population group			
Jews	550 (50.2)		
Arabs	546 (49.8)		
Socioeconomic status (cluster) and population group	All	Jews	Arabs
Low (Clusters 1–2)	491 (44.8)	89 (18.1)	402 (81.9)
High (Clusters 3–10)	605 (55.2)	461 (61.7)	144 (38.3)

**Table 2:** Analysis of factors associated with IgG anti-*S. sonnei* LPS levels.

Factors	N	GM	95%CI	N (%), Cutoff ≥ 4.5 EUs	*p* Value *	Unadjusted OR (95% CI)	*p* Value #
Age					<0.001		
<1 year	476	0.12	0.10–0.14	5 (1.1)		Reference	
0–3 months	118	0.58	0.45–0.75	5 (4.2)			
4–11 months	358	0.07	0.06–0.09	0 (0.0)			
1–4 years	320	0.33	0.27–0.40	24 (7.5)		7.64 (2.88–20.24)	<0.001
5–14 years	200	1.45	1.17–1.80	48 (24.0)		29.75 (11.63–76.08)	<0.001
15–19 years	100	2.37	1.77–3.18	36 (36.0)		52.99 (20.06–139.94)	<0.001
Sex					0.041		
Males	575	0.30	0.25–0.35	49 (8.5)		Reference	-
Females	521	0.39	0.32–0.46	64 (12.3)		1.50 (1.02–2.23)	0.042
Population group					<0.001		
Jews	550	0.27	0.22–0.31	40 (7.3)		Reference	-
Arabs	546	0.43	0.36–0.51	73 (13.4)		1.97 (1.31–2.95)	0.001
Socioeconomic status					<0.001		
Clusters 1–2	491	0.47	0.39–0.57	78 (15.9)		3.08 (2.02–4.67)	<0.001
Clusters 3–10	605	0.25	0.22–0.30	35 (5.8)		Reference	-

GM ELISA units of a standard (EUs); OR: odds ratio; CI: Confidence Interval. * chi-square for proportions; # chi square for OR.

**Table 3:** Multivariable logistic regression models of factors associated with the prevalence of IgG anti-*S. sonnei* LPS ≥ 4.5 EUs.

Factors	Model 1		Model 2		Model 3	
	Adjusted OR (95% CI)	*p* Value	Adjusted OR (95% CI)	*p* Value	Adjusted OR (95% CI)	*p* Value
Age						
<1 year	Reference	-	Reference	-	Reference	-
1–4 years	7.49 (2.82–19.88)	<0.001	6.72 (2.53–17.87)	<0.001	6.71 (2.52–17.86)	<0.001
5–14 years	30.06 (11.72–77.13)	<0.001	27.71 (10.79–71.18)	<0.001	27.68 (10.77–71.13)	<0.001
15–19 years	54.77 (20.62–145.49)	<0.001	48.62 (18.28–129.31)	<0.001	48.62 (18.28–129.30)	<0.001
Sex						
Male	Reference	0.087	Reference	0.086	Reference	0.086
Female	1.45 (0.95–2.24)		1.46 (0.95–2.25)		1.46 (0.95–2.25)	
Population group						
Jews	Reference	<0.001	-	-	Reference	0.942
Arabs	2.20 (1.42–3.42)		-		1.03 (0.52–2.02)	
Socioeconomic status						
Clusters 1–2	-	-	2.79 (1.78–4.38)	<0.001	2.73 (1.37–5.46)	0.004
Clusters 3–10	-	-	Reference		Reference	

GM ELISA units of a standard (EUs); OR: odds ratio; CI: Confidence Interval.

## Data Availability

The original contributions presented in the study are included in the article/Supplementary Material, further inquiries can be directed to the corresponding author.

## Supplementary Materials

The following supporting information can be downloaded at: https://www.mdpi.com/article/10.3390/vaccines12030239/s1, Figure S1: Serum IgG anti-*S. sonnei* LPS antibodies levels and incidence of *S. sonnei* shigellosis in Israeli Jewish and Arab children and adolescents; Figure S2: Serum IgG anti-*S. sonnei* LPS antibodies levels and incidence of *S. sonnei* shigellosis in Israeli Jewish and Arab infants; Figure S3: Levels of serum IgG anti-*S. sonnei* LPS antibodies and incidence of *S. sonnei* shigellosis in Israeli children and adolescents from the South of Israel; Figure S4: Serum IgG anti-*S. sonnei* LPS antibodies levels and incidence of *S. sonnei* shigellosis in Israeli infants from the South of Israel; Table S1: Levels of serum IgG anti-*S. sonnei* LPS antibodies expressed as GM ELISA units of a standard (EUs) and proportion of individuals with IgG anti-*S. sonnei* LPS levels ≥ 4.5 EUs in 1,096 children and adolescents; Table S2: Levels of serum IgG anti-*S. sonnei* LPS antibodies, expressed as GM ELISA units of a standard (EUs) and proportion of individuals with IgG anti-*S. sonnei* LPS levels ≥ 4.5 EUs in 476 infants; Table S3: Levels of serum IgG anti-*S. sonnei* LPS antibodies, expressed as GM ELISA units of a standard (EUs) in groups of Jewish and Arab children and adolescents and a proportion of Jewish and Arab children and adolescents with IgG anti-*S. sonnei* LPS levels ≥ 4.5 EUs; Table S4: Levels of serum IgG anti-*S. sonnei* LPS antibodies, expressed as GM ELISA units of a standard (EUs), in Jewish and Arab infants; Table S5: GMT of serum IgG anti-*S. sonnei* LPS antibodies in 1,096 children and adolescents (corresponding to GM EUs levels in Table S1); Table S6: GMT of serum IgG anti-*S. sonnei* LPS antibodies in 476 infants (corresponding to GM EUs levels in Table S2); Table S7: Spearman’s correlations between GM or prevalence of IgG anti-*S. sonnei* LPS levels according to various threshold EUs values and incidences of *S. sonnei* shigellosis determined in 8 age groups.
