# Supplementary material for: IgG Serum Antibodies to Shigella sonnei Lipopolysaccharide Are Inversely Associated with the Incidence of Culture-Proven S. sonnei Shigellosis in Israeli Children and Adolescents
Source: Vaccines (Basel). 2024 Feb 25;12(3):239. doi: 10.3390/vaccines12030239 (PMC10974432; doi:10.3390/vaccines12030239)
Supplement: Supplementary file 1 [file vaccines-12-00239-s001.zip › vaccines-2836635-supplementary material.pdf]

Supplementary material

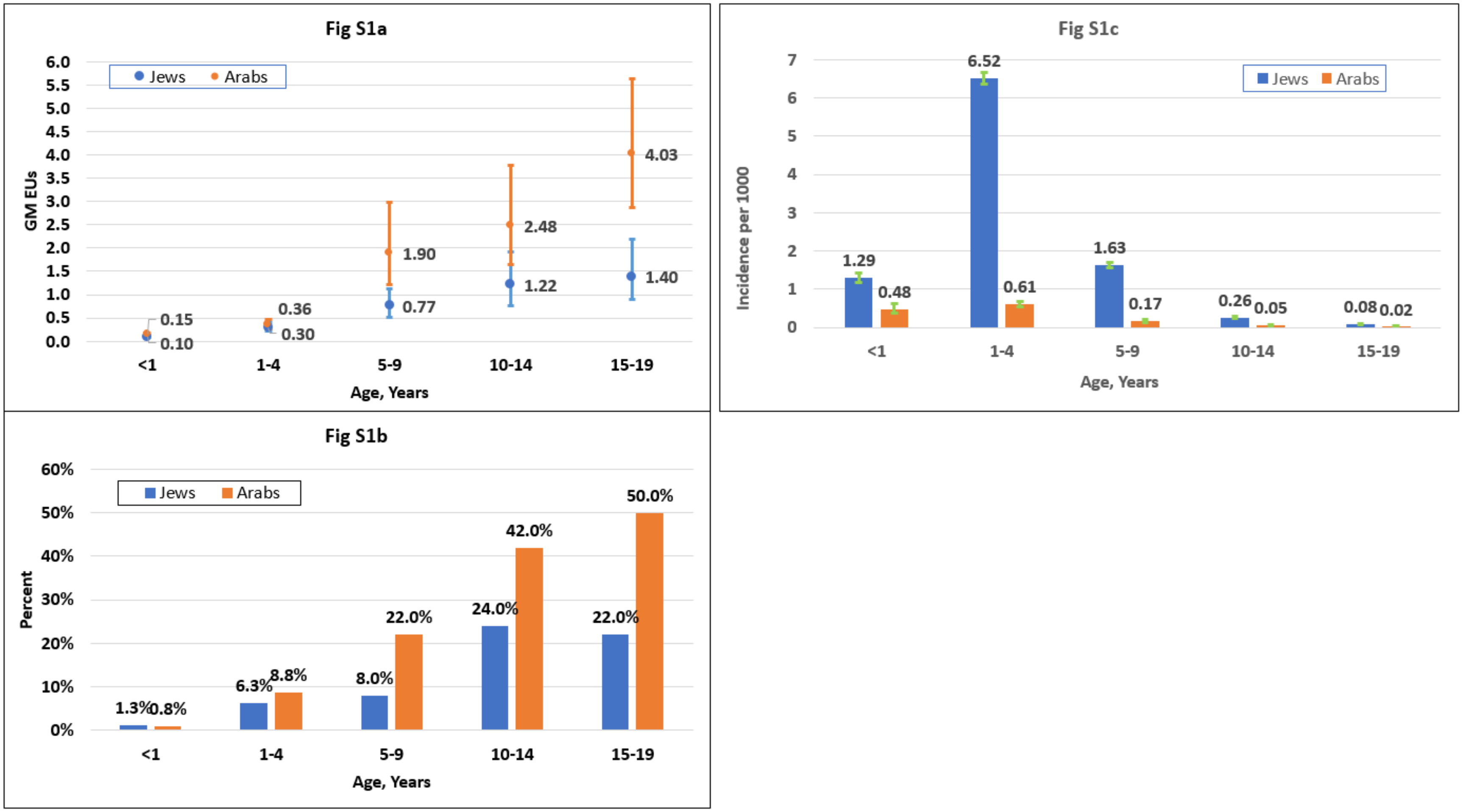

Figure S1: a. Levels of serum IgG anti-*S. sonnei* LPS antibodies, expressed as GM ELISA units of a standard (EUs), in ascending age groups of Israeli Jewish and Arab children and adolescents. Vertical lines indicate 95% CI. (Total Jewish children and adolescents, n=550; Total Arab children and adolescents, n=546) **b.** Proportion of Israeli Jewish and Arab children and adolescents with IgG anti-*S. sonnei* LPS levels ≥ 4.5 EUs (Total Jewish children and adolescents, n=550; Total Arab children and adolescents, n=546).

c. Average annual incidence per 1000 of *S. sonnei* shigellosis in Jewish and Arab children and adolescents stratified by age during 2008-2015 (based on 10,293 isolates in Jews and 514 isolates in Arabs for which the population group of patients was known). Vertical lines indicate 95% CI.

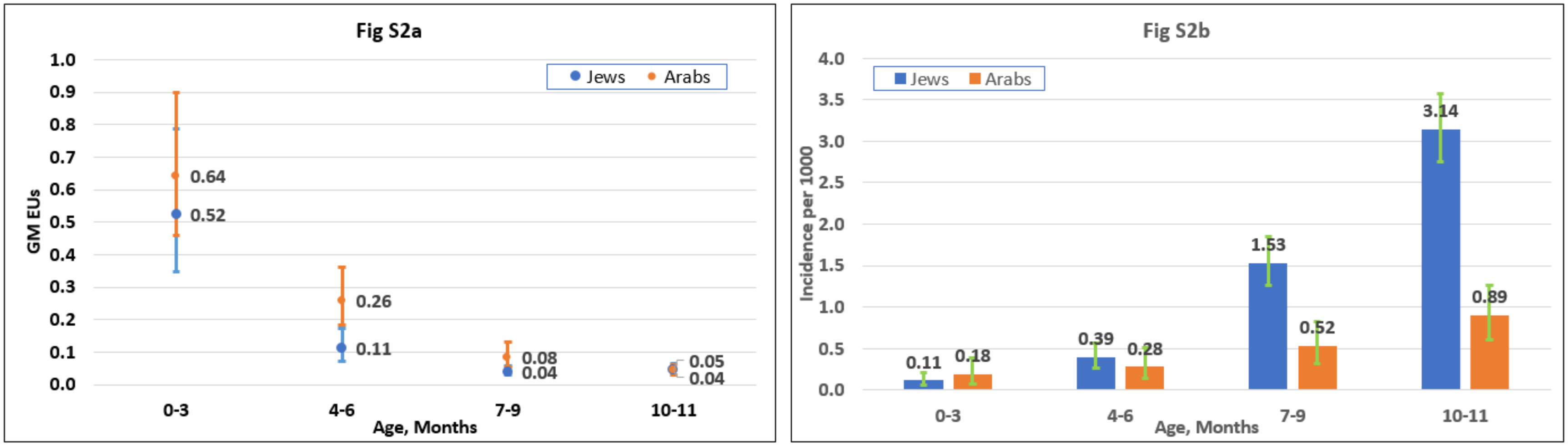

Figure S2 a. Levels of serum IgG anti-*S. sonnei* LPS antibodies, expressed as GM ELISA units of a standard (EUs), in Israeli Jewish and Arab infants. Vertical lines indicate 95% CI. **b**. Average annual incidence per 1000 of *S. sonnei* shigellosis in Israeli Jewish and Arab infants during 2008-2015 (based on 368 isolates in Jews and 61 isolates in Arabs for which the population group of patients was known). Vertical lines indicate 95% CI.

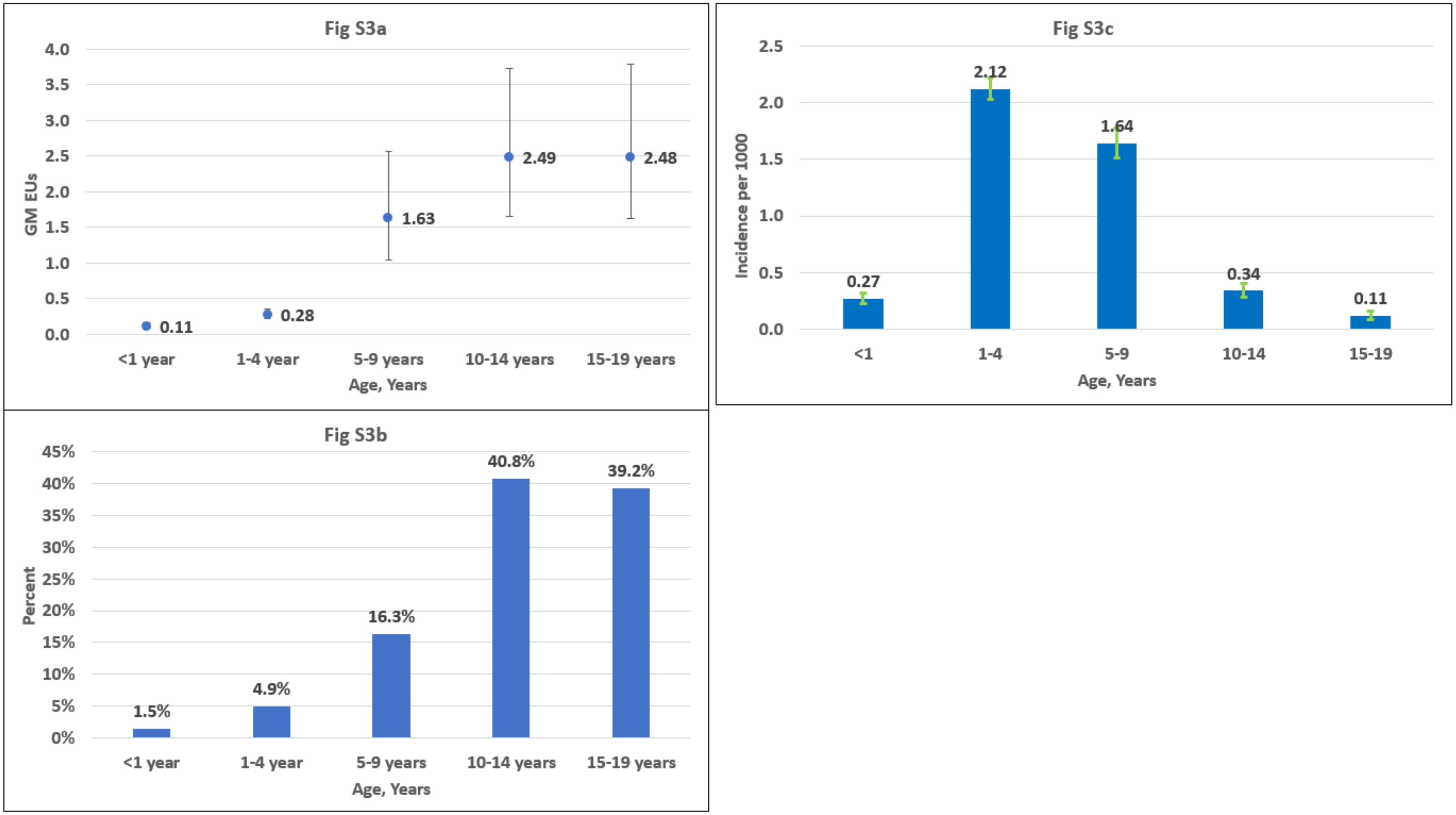

Figure S3. a. Levels of serum IgG anti-*S. sonnei* LPS antibodies expressed as GM ELISA units of a standard (EUs) in ascending age groups of 467 Israeli children and adolescents from the South of Israel. Vertical lines indicate 95% CI. **b.** Proportion of individuals with IgG anti-*S. sonnei* LPS levels ≥ 4.5 EUs in ascending age groups of 467 Israeli children and adolescents from the South of Israel.

c. Average annual incidence per 1000 of *S. sonnei* shigellosis in the population served by the sentinel laboratory in the South of Israel stratified by the corresponding age groups, during 2008-2015 (based on 2762 *S. sonnei* isolates). Vertical lines indicate 95% CI.

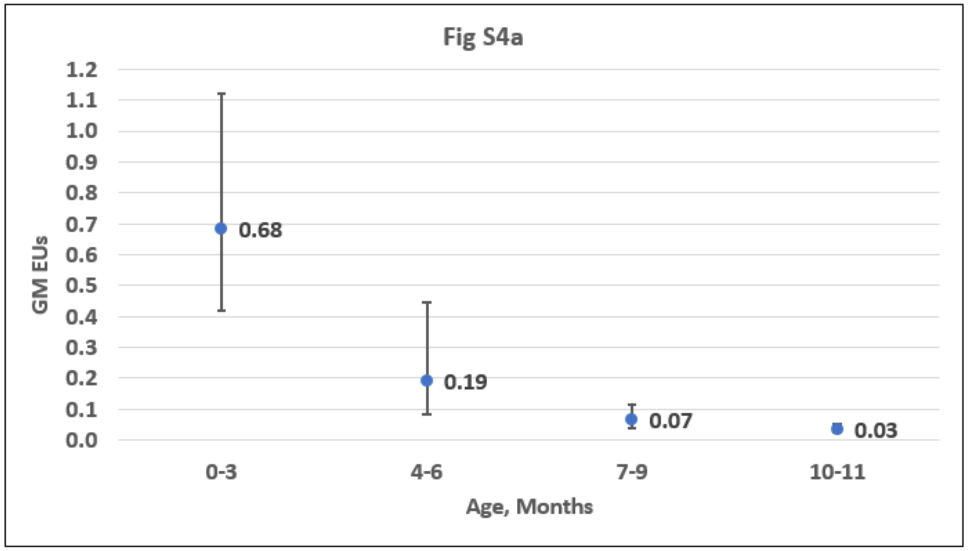

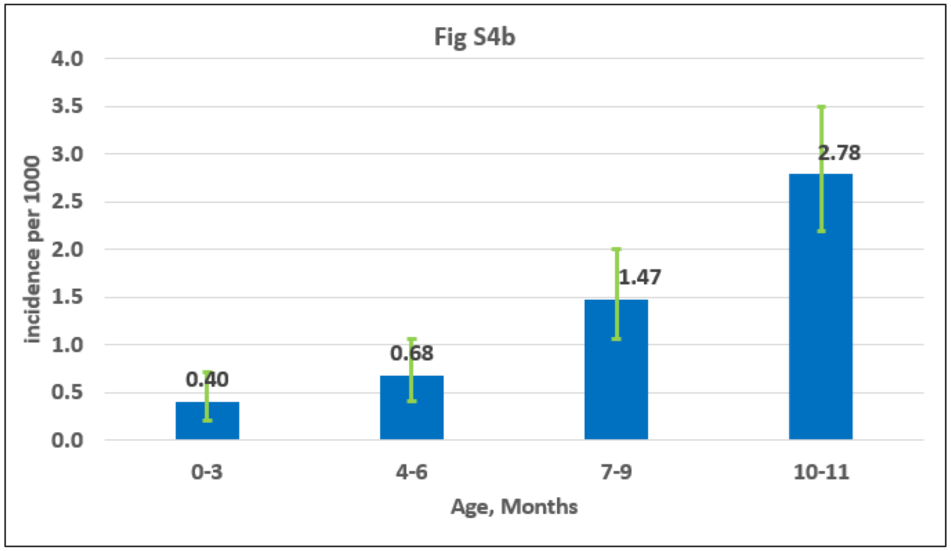

Figure S4 a. Levels of serum IgG anti-*S. sonnei* LPS antibodies, expressed as GM ELISA units of a standard (EUs), in 135 infants from the South of Israel. Vertical lines indicate 95% CI. **b**. Average annual incidence per 1000 of *S. sonnei* shigellosis in the population of infants served by the sentinel laboratory in the South of Israel stratified by the corresponding age groups, during 2008-2015 (based on 134 *S. sonnei* isolates). Vertical lines indicate 95% CI.

Table S1: Levels of serum IgG anti-*S. sonnei* LPS antibodies expressed as GM ELISA units of a standard (EUs) and proportion of individuals with IgG anti-*S. sonnei* LPS levels ≥ 4.5 EUs in 1096 children and adolescents.

Age, Years	N	GM EUs	95% Confidence Interval for GM EUs		% of anti-*S. sonnei* LPS levels ≥ 4.5 EUs	95% Confidence Interval for % of anti- *S.sonnei* LPS levels ≥ 4.5 EUs	
			Lower Bound	Upper Bound		Lower Bound	Upper Bound
**<1**	476	0.123	0.104	0.144	1.1%	0.3%	2.4%
**1-4**	320	0.326	0.267	0.399	7.5%	4.9%	11.0%
**5-9**	100	1.209	0.892	1.639	15.0%	8.7%	23.5%
**10-14**	100	1.742	1.275	2.379	33.0%	23.9%	43.1%
**15-19**	100	2.373	1.769	3.183	36.0%	26.6%	46.2%

Table S2: Levels of serum IgG anti-*S. sonnei* LPS antibodies, expressed as GM ELISA units of a standard (EUs) and proportion of individuals with IgG anti-*S. sonnei* LPS levels ≥ 4.5 EUs in 476 infants

Age, Months	N	GM EUs	95% Confidence Interval for GM EUs		% of anti-*S. sonnei* LPS levels ≥ 4.5 EUs	95% Confidence Interval for % of anti- *S.sonnei* LPS levels ≥ 4.5 EUs	
			Lower Bound	Upper Bound		Lower Bound	Upper Bound
**0-3**	118	0.578	0.445	0.751	4.2%	1.4%	9.6%
**4-6**	116	0.168	0.127	0.223	-	-	-
**7-9**	111	0.056	0.042	0.074	-	-	-
**10-11**	131	0.045	0.035	0.057	-	-	-

Table S3: Levels of serum IgG anti-*S. sonnei* LPS antibodies, expressed as GM ELISA units of a standard (EUs) in groups of Jewish and Arab children and adolescents and a proportion of Jewish and Arab children and adolescents with IgG anti-*S. sonnei* LPS levels ≥ 4.5 EUs

Age, Years	N	GM EUs	95% Confidence Interval for GM EUs		% of anti-*S. sonnei* LPS levels ≥ 4.5 EUs	95% Confidence Interval for % of anti- *S.sonnei* LPS levels ≥ 4.5 EUs	
			Lower Bound	Upper Bound		Lower Bound	Upper Bound
**Jews:**							
**<1**	240	0.102	0.081	0.128	1.3%	0.3%	3.6%
**1-4**	160	0.296	0.221	0.395	6.3%	3.0%	11.2%
**5-9**	50	0.770	0.524	1.132	8.0%	2.2%	19.2%
**10-14**	50	1.221	0.774	1.927	24.0%	13.1%	38.2%
**15-19**	50	1.398	0.895	2.183	22.0%	11.5%	36.0%
**Arabs:**							
**<1**	236	0.148	0.118	0.186	0.8%	0.1%	3.0%
**1-4**	160	0.360	0.272	0.477	8.8%	4.9%	14.3%
**5-9**	50	1.900	1.213	2.974	22.0%	11.5%	36.0%
**10-14**	50	2.485	1.637	3.772	42.0%	28.2%	56.8%
**15-19**	50	4.029	2.880	5.636	50.0%	35.5%	64.5%

Table S4: Levels of serum IgG anti-*S. sonnei* LPS antibodies, expressed as GM ELISA units of a standard (EUs), in Jewish and Arab infants.

Age, Months	N	GM EUs	95% Confidence Interval for GM EUs	
			Lower Bound	Upper Bound
**Jews:**				
**0-3**	60	0.523	0.348	0.785
**4-6**	60	0.113	0.073	0.174
**7-9**	60	0.040	0.029	0.056
**10-11**	60	0.045	0.031	0.066
**Arabs:**				
**0-3**	58	0.642	0.458	0.899
**4-6**	56	0.258	0.183	0.362
**7-9**	51	0.084	0.053	0.132
**10-11**	71	0.044	0.031	0.062

Table S5: GMT* of serum IgG anti-*S. sonnei* LPS antibodies in 1096 children and adolescents (corresponding to GM EUs levels in Table S1).

Age, Years	N	GMT	95% Confidence Interval	
			Lower Bound	Upper Bound
**<1**	476	24.76	20.92	29.32
**1-4**	320	68.74	55.71	84.81
**5-9**	100	269.99	196.56	370.84
**10-14**	100	395.15	285.34	547.21
**15-19**	100	545.78	401.63	741.67

*GMT=Geometric Mean Titer

Table S6: GMT* of serum IgG anti-*S. sonnei* LPS antibodies in 476 infants (corresponding to GM EUs levels in Table S2).

Age, Months	N	GMT	95% Confidence Interval	
			Lower Bound	Upper Bound
**0-3**	118	124.99	95.09	164.29
**4-6**	116	34.40	25.57	46.27
**7-9**	111	10.96	8.16	14.70
**10-11**	131	8.60	6.63	11.15

*GMT=Geometric Mean Titer

Table S7: Spearman's correlations between GM or prevalence of IgG anti-*S. sonnei* LPS levels according to various threshold EUs values and incidences of *S. sonnei* shigellosis determined in 8 age groups.

	n	Incidences 2008-2015	
		Spearman's rho	p-value
**GM of serum IgG anti-*S. sonnei* LPS antibodies**	8	-0.762	**0.028**
**% with IgG anti-*S. sonnei* LPS levels ≥ 3 EUs**	8	-0.643	0.086
**% with IgG anti-*S. sonnei* LPS levels ≥ 3.5 EUs**	8	-0.643	0.086
**% with IgG anti-*S. sonnei* LPS levels ≥ 4.5 EUs**	8	-0.586	0.127
**% with IgG anti-*S. sonnei* LPS levels ≥ 5.5 EUs**	8	-0.586	0.127
**% with IgG anti-*S. sonnei* LPS levels ≥ 6 EUs**	8	-0.577	0.134
